# Food price seasonality in Africa: Measurement and extent

**DOI:** 10.1016/j.foodpol.2016.09.016

**Published:** 2017-02

**Authors:** Christopher L. Gilbert, Luc Christiaensen, Jonathan Kaminski

**Affiliations:** a SAIS Bologna Center, Johns Hopkins University, Bologna, Italy; b World Bank, Brussels, Belgium; c Geneva, Switzerland

## Abstract

Everyone knows about seasonality. But what exactly do we know? This study systematically measures seasonal price gaps at 193 markets for 13 food commodities in seven African countries. It shows that the commonly used dummy variable or moving average deviation methods to estimate the seasonal gap can yield substantial upward bias. This can be partially circumvented using trigonometric and sawtooth models, which are more parsimonious. Among staple crops, seasonality is highest for maize (33 percent on average) and lowest for rice (16½ percent). This is two and a half to three times larger than in the international reference markets. Seasonality varies substantially across market places but maize is the only crop in which there are important systematic country effects. Malawi, where maize is the main staple, emerges as exhibiting the most acute seasonal differences. Reaching the Sustainable Development Goal of Zero Hunger requires renewed policy attention to seasonality in food prices and consumption.

## 1 Introduction

It is well-known that agricultural prices vary across seasons, typically peaking just before the harvest, and dropping substantially immediately thereafter. Despite this, there exists little systematic research on the extent of this seasonal variation across food commodities, countries, or markets within countries. The only comprehensive analysis that systematically applies the same methodology across commodities and countries is Sahn and Delgado (1989). This is by now somewhat dated. The consequence is that, although “we all know about seasonality”, it is very unclear precisely what it is we know.^3^

Knowing the extent of food price seasonality matters for a number of reasons. First, when food prices display high seasonality, so may also be dietary intake and nutritional outcomes, with episodes of nutritional deficiencies during the first 1000 days of life particularly detrimental for cognitive development and future earnings (Dercon and Portner, 2014). The 2015 adoption of Sustainable Development Goal II of Zero Hunger^4^ adds pertinence.^5^ When production is cyclical, some seasonality in prices is normal; intertemporal arbitrage is needed and storage costs ensue, which drive a wedge between prices before and after the harvest.^6^ This gap can be compounded by poorly integrated markets and trade restrictions, market power along the marketing chain, and sell-low, buy-back-high behavior among liquidity and credit constrained households (Stephens and Barrett, 2011). They can push up the seasonal price gap well beyond the levels expected in settings with well-functioning markets.

Excess seasonality in prices may further translate into seasonal variation in dietary intake and nutrition, for example, when households are credit constrained or ill-equipped with other coping strategies, as has been documented in Ethiopia (Dercon and Krishnan, 2000), Bangladesh (Khandker, 2012), and Tanzania (Kaminski et al., 2016).^7^ Moderation of seasonal price variation (for example through facilitation of storage or access to credit) could then be a way to increase overall food and nutrition security.

A second reason for refocusing attention to food price seasonality relates to the sharply increased volatility of world food prices in the immediate aftermath of the 2007–08 world food crisis (Gilbert and Morgan, 2010, Gilbert and Morgan, 2011) although volatility levels appear to have dropped back since that time (Minot, 2014). This volatility was transmitted to a greater or lesser extent to food prices in developing countries and attracted considerable government attention (Galtier and Vindel, 2012, World Bank, 2012, Ceballos et al., 2015). Food price volatility arises from both international and domestic shocks to production (harvest shocks) or consumption (changes in purchasing power). However, seasonality (i.e. known fluctuations) also contributes to price volatility (especially domestically) and would require different policy instruments to address it. Little is known on the extent of this possibility.

The third reason relates to the measurement and analysis of poverty (the focus of the first Sustainable Development Goals). Poverty measurement relies heavily on food expenditure information which is typically collected only once for each household during at a particular point during the year (with a 7–30 day recall period). The annual expenditures measures derived from these surveys will be incorrect when food price seasonality is substantial and not corrected for, as is mostly the case in current practice (Muller, 2002, Van Campenhout et al., 2015).

The seasonal gap—the difference between the high price immediately prior to the harvest and the low price following the harvest, averaged across years—is the standard measure used to measure the extent of seasonality. It is common to estimate this gap from a (monthly) dummy variables regression on trend-adjusted prices or simply from the (monthly) mean price deviation around a moving average trend (Goetz and Weber, 1986, Chapter IV).

Using Monte Carlo simulations, this paper shows that, when samples are short (5–15 years), these approaches can seriously overestimate the extent of seasonality, especially when there is either little seasonality or where the seasonal pattern is poorly defined. Although the coefficients of individual monthly dummy variables, or the monthly price averages, are individually unbiased, the seasonal gap, which is obtained as the difference between the maximum and the minimum dummy coefficient, each identified from the data, is upwardly biased. This problem has hitherto not been noted despite the relatively short samples typically used in the development literature on seasonality.

It is shown that the problem can be mitigated by using trigonometric or sawtooth models. These more parsimonious models impose some structure on the nature of seasonality, thereby substantially reducing the number of parameters to be estimated and providing more observations per estimated parameter. This substantially reduces the upward bias in the estimated gap. When there is more than one season, which is less common, the dummy variable approach may still perform better, because it is more flexible.

To select the preferred specification and minimize the upward bias when estimating the seasonal gap, a three step procedure is advanced. Systematically applying this three step approach, the extent of price seasonality is measured by market place (typically major provincial centers) for 13 food commodities in seven Sub-Saharan African countries, or a total of 1053 market place-commodity pairs. In each case, there are between six and 13 years of monthly data depending on the country, market place and commodity.

The findings indicate that seasonality in African food markets remains sizeable. The seasonal gap is highest among vegetables (60.8 percent for tomatoes) and fruits, and lowest among commodities which are produced throughout the year (eggs) and/or whose harvest is not season bound (cassava). Among staple grains, seasonality is highest for maize (33.1 percent on average) and lowest for rice (16.6 percent). These gaps are two and a half to three times higher than on the international reference markets, pointing to substantial excess seasonality. While excess seasonality is observed in virtually all the maize and rice markets studied, there is wide heterogeneity within and across countries. Seasonality is especially high in Malawi, where maize is also the main staple, causing a double seasonality burden for most households.

In what follows, Section 2 sets the stage by reviewing general considerations on the data, seasonality metrics and the overall estimation approach. Section 3 looks at the commonly used methods for estimating the seasonal gap and shows that these can result in upwardly biased estimates when data samples are short. The performance of alternative and more parsimonious seasonality models is examined in Section 4. Section 5 introduces the price data from the thirteen commodities and seven African countries examined here and discusses the findings. Section 6 concludes.

## 2 Material, metrics and method – general considerations

Many developing country governments publish monthly prices for staple food commodities for major locations in their territories. These prices are obtained by sending observers to markets in these locations, who record the prices at which the different commodities are transacted. It is unclear how much intra-month averaging is undertaken, but at least for some countries (e.g. Uganda), the monthly prices derive from weekly observations. Much of this price information results from the FEWSNET initiative, supported by USAID, and the FAO’s GIEWSNET initiative.

Three features of these price data stand out. First, the price data collection initiatives are relatively recent so that the time series available are usually short. Second, in many of the price series, the frequent occurrence of missing observations compounds the short duration of the series. Gaps may arise for example because the observers did not see transactions in the foods in question when they visited the markets. In some other instances, prices are missing for all locations in a particular month suggesting an administrative explanation. Finally, in most countries, only a small number of (mainly urban) locations (five to fifteen) are covered, though some governments (Malawi in our sample) attempt to be more comprehensive. These features of the data are important to keep in mind when measuring seasonality in developing countries. They also caution against overgeneralization based on a small number of market locations within countries, as seasonality will prove to differ substantially from place to place.

In agriculture, seasonality measures attempt to capture the part of the intra-annual variability of the monthly observations that is specifically related to the crop cycle. The simplest case is that of a subsistence crop with a single annual harvest and for which imports and exports are unimportant within a wide price band. The price of such a commodity will be lowest immediately after the harvest and will then rise steadily until the following harvest to reflect (at a minimum) storage and deterioration costs. The most widely used seasonality measure for such products is the seasonal gap (also used here), which is the expected (or average) fall in price over the pre- and post-harvest period.^8^

The basic structural representation of seasonality in a price series considers three components: trend, seasonal factors and irregular variation:

(1) $$p_{\mathrm{ym}}=\mu_{\mathrm{ym}}+s_{m}+\varepsilon_{\mathrm{ym}}$$

where *p_ym_* is the logarithm of the food price in month *m* of year *y*, $\mu_{\mathrm{ym}}$ is the trend, $s_{1}\text{,}\ldots \text{,}s_{12}$ are a set of twelve seasonal factors satisfying $\sum_{j=1}^{12}s_{j}=0$ and $\varepsilon_{\mathrm{ym}}$ is a disturbance.^9^ In this framework, the standard measure of the seasonal gap is the difference between the highest and the lowest seasonal factor:

(2) $$\mathrm{gap}=\max s_{m}-\min s_{m}$$

There are three issues: the specification of the trend component $\mu_{\mathrm{ym}}$, the estimation of the seasonal factors $s_{1}\text{,}\ldots \text{,}s_{12}$, and the treatment of missing values. The choice of trend specification affects flexibility in dealing with missing values. These two issues are discussed together. The simplest trend estimation procedure is to specify a linear trend. The seasonal factors can be estimated from the regression:

(3) $$p_{\mathrm{ym}}=\kappa +\gamma t+\overset{11}{\underset{j=1}{\sum }}\delta_{j}z_{\mathrm{mj}}+\varepsilon_{\mathrm{ym}}$$

where the trend t=12∗(y-1)+m and *z_mj_* is the dummy variable defined by $z_{\mathrm{mj}}=\left\{\begin{array}{cc} 1 & j=m\\ 0 & j\;\neq \;m \end{array}\right)$. Normalizing δ_12_ = 0 gives seasonal factors:

(4) $$\begin{array}{cc} s_{m}=\delta_{m}-\frac{1}{12}\overset{12}{\underset{j=1}{\sum }}\delta_{j} & \left(m=1\text{,}\ldots \text{,}12\right) \end{array}$$

Sahn and Delgado (1989) adopt this approach.

The linear trend approach assumes that prices are trend stationary, i.e. that they revert to a deterministic trend. However, economic theory does not provide any basis to suppose that food price trends are constant. One way to allow for a variable trend is to estimate the trend as a centered moving average, which can vary from month to month:

(5) $$\mu_{\mathrm{ym}}=\frac{1}{12}\left[\overset{5}{\underset{j=-5}{\sum }}p_{y\text{,}m+j}+\frac{1}{2}(p_{y\text{,}m+6}+p_{y\text{,}m-6})\right]$$

Using this approach, seasonal factors can be estimated as average deviations from the detrended price series so that

(6) $$\begin{array}{cc} s_{m}=\frac{1}{Y}\overset{Y}{\underset{y=1}{\sum }}(p_{\mathrm{ym}}-\mu_{\mathrm{ym}}) & \left(m=1\text{,}\ldots \text{,}12\right) \end{array}$$

This is the approach adopted in Allen (1954) and Goetz and Weber (1986).

While straightforward to apply and widely used in the agricultural and development literature, this moving average deviation (MAD) procedure also comes with important disadvantages. First, calculation of the moving average price trend sacrifices the initial and final six months of the dataset. If the available sample is short, this can be a major loss. Second, estimation of the moving average price trend requires interpolation of the missing data points. In the absence of clear conceptual guidance on the appropriate information base for interpolation, this poses a concern.^10^ Third, the procedure of taking deviations from the moving average trend induces a complicated moving average error into the disturbance term associated with the price deviations. This does not affect the calculation of seasonal factors but will invalidate standard statistical inference.

The alternative approach, which we adopt in what follows, is to suppose that the price trend is stochastic. Even if price series are non-trend-stationary, they will generally be difference stationary (Nelson and Kang, 1984). This yields the stochastic trend model (Stock and Watson, 2003, chapter 12). Like the MAD procedure, the stochastic trend model allows for a trend which varies over time, albeit with a constant annual increment. It sets

(7) $$\mu_{\mathrm{ym}}=\mu_{y\text{,}m-1}+\gamma +\nu_{\mathrm{ym}}$$

As in the linear trend model (3), γ is the monthly trend increment. Differencing Eq. (1) and substituting Eq. (7) yields

(8) $$\Delta p_{\mathrm{ym}}=\gamma +\Delta s_{m}+u_{\mathrm{ym}}$$

where *u_ym_* is a compound error term. The estimating equation becomes

(9) $$\Delta p_{\mathrm{ym}}=\gamma +\overset{11}{\underset{j=1}{\sum }}\delta_{j}\Delta z_{\mathrm{mj}}+u_{\mathrm{ym}}$$

where the differenced dummies^11^ Δ*z_mj_* (*j* = 1,…,11) are defined by $\Delta z_{\mathrm{mj}}=\left\{\begin{array}{cc} \begin{array}{c} 1\\ -1\\ 0 \end{array} & \begin{array}{c} m=j\\ m=j-1\\ \text{otherwise} \end{array} \end{array}\right)$.

The approach set out in Eq. (9) has important advantages over the MAD procedure. First, only a single observation is lost through differencing compared with twelve in the MAD procedure. Second, there is no requirement for interpolation over gaps.^12^ If there is a gap of *k* months prior to observation (*y*,*m*), Eq. (9) can be replaced by

(10) $$\Delta_{k}p_{\mathrm{ym}}=p_{\mathrm{ym}}-p_{y\text{,}m-k-1}=k\gamma +\overset{k-1}{\underset{i=0}{\sum }}s_{m-i}+w_{\mathrm{ym}}$$

where *w_ym_* is a new compound error term.^13^

## 3 Bias in seasonal gap estimates

Regression on a set of constants, as in Eqs. (3), (9), yields unbiased and consistent coefficient estimates. It follows that the estimated seasonal factors $s_{1}\text{,}\ldots \text{,}s_{12}$ are also unbiased. If we know, a priori, that the seasonally high pre-harvest price is in month *hi* and the seasonally low post-harvest price in month *lo*, then the seasonal gap as measured by $\mathrm{gap}=s_{\mathrm{hi}}-s_{\mathrm{lo}}$ will also be unbiased. In this circumstance, the dummy variables estimator of the seasonal gap works well. However, the exact timing of seasonal peaks and troughs varies across crop-location pairs, even within countries. Even knowledgeable observers, but especially the analyst sitting in London, Paris or Washington, may well be unfamiliar with harvest patterns in all locations and may wish to estimate these from the price data.

In these circumstances, the analyst will use the gap estimate defined by Eq. (2), $\mathrm{gap}=\max s_{m}-\min s_{m}\text{.}$ This is biased upwards, even though consistent, when identified from the data. Intuitively, while the empirical estimates of the seasonal factors (or monthly dummies) are each unbiased, each empirical estimate of a seasonal factor represents a draw from a distribution, which usually deviates slightly from its true point value. As a result, by taking each time the maximum and minimum values of all seasonal factors, the gap will be overestimated.

In statistical terms, while a linear transformation of two unbiased statistics remains unbiased, this does not hold when the transformation is non-linear. The estimated gap measure defined by Eq. (2) is a non-negative and a nonlinear function of the seasonal factors and therefore (upwardly) biased.^14^ By contrast, the gap measure $s_{\mathrm{hi}}-s_{\mathrm{lo}}$ for known peak and trough months is a difference between two unbiased statistics and will itself be unbiased. In a particular sample, this gap measure for a known harvest month may either be positive or negative, although it is likely to be positive. The difference between the maximum and the minimum, $\max s_{m}-\min s_{m}$, is necessarily positive.

The problem arises because the peak and trough months identified in any particular sample may differ from those defined by the harvest pattern. This misrepresentation is more likely in short samples and with data where the harvest cycle contributes only a small proportion of total price variation. To appreciate the conceptual and empirical importance of this insight, consider the extreme case in which there is no seasonality (i.e. no price difference between the pre- and postharvest months). Picking the largest and the smallest monthly estimates necessarily yields a positive seasonal gap, suggesting spurious evidence of seasonality. This is despite the fact that each of the seasonal factor estimates is unbiased.

In sum, bias in the dummy variables gap estimate arises from three separate factors which interact with each other:

• peak and trough months are identified from the data;
• the estimated gap is a nonlinear function of the (unbiased) dummy variable coefficients;
• the small number of observations typically used to estimate the coefficients of the peak and trough month dummy variables. (What is relevant here is the number of years of data in the sample, not the number of monthly observations).

In samples in which the peak and trough months are clearly defined or the gap is large, it is unlikely that the procedure will make an incorrect peak-trough identification or that the estimated coefficient of the trough month dummy will exceed that of the peak month dummy (an apparent “seasonal reversal”). A small number of annual observations will yield an imprecise (high variance) gap estimate but this estimate is as likely to be too low as too high. In the opposite case, in which the actual gap is low and/or the peak and trough months are poorly defined, the dummy variables gap estimator will select those peak and trough months which happen, in the sample available, to give the highest gap estimate. The estimator will be consistent since, given a sufficiently long sample, the correct peak-trough identification will be made and the probability of a seasonal reversal will approach zero. However, with the sample sizes typically available in an African context, the probability of bias is high.

To illustrate, two sets of Monte Carlo experiments are reported. The first set of experiments (Table 1) estimates the seasonal gaps using the dummy variable regression (9), based on a stochastic trend model. The second set (Table 2) uses the MAD procedure where the moving average trend estimate is defined by Eq. (5) and estimation using Eq. (6). In each set of experiments, the data were generated according to Eq. (9). The disturbances *u_ym_* were are independently distributed $N(0\text{,}0.15^{2})$.

There are three sub-cases:

(a) Columns 1–3: Data generated with no seasonality.
(b) Columns 4–6. Data generated with a clear and regular sawtooth (i.e. non-symmetric) seasonal pattern. On average, prices fall by 10 percent in each of January and February and rise by 2 percent in the remaining eleven months implying a 20 percent gap (more on sawtooth seasonal patterns below).
(c) Columns 7–9. Data generated by a diffuse and less well-defined seasonal pattern. On average, prices fall by 4 percent in each of January and February and rise by 0.8 percent in the remaining ten months implying a 4 percent gap. However, one year in five, the harvest is retarded by one month such that the price falls in February instead of January. Taking into account the fact that January prices continue to rise one year in five, this gives a seasonal gap of 8.16 percent.

In each case, four samples are considered, of length 5, 10, 20 and 40 years of monthly data. The results reported are based on 100,000 replications. The tables also report the average regression *R^2^* (i.e. share of the price variation in the sample on average “explained” by the seasonal factors) and the proportion of simulations in which the regression *F* statistic rejects the hypothesis of no seasonality.

The dummy variable estimates for the stochastic trend model (Table 1) are considered first.

(a) When there is no seasonality, the gap measure shows substantial upward bias. The estimated gap is 21 percent and 15 percent using five and ten years of data respectively. The *R*^2^ statistics indicate that around 19 percent and 9 percent of the sample price variation respectively are “explained” by seasonality. However, the *F* tests correctly show that, at the 5 percent level, only around 5 percent of the estimates reject the null of no seasonality.
(b) In the case of clear seasonality, the dummy variables gap estimator remains upwardly biased, but by much less (8 percent on five years data and 4 percent on ten years data). Unsurprisingly, the *R*^2^ statistics are higher than in the no seasonality case but with ten years data, the null of no seasonality is only rejected in approaching half the cases.
(c) The third case, diffuse seasonality, generates intermediate results. The bias is substantial in short samples (14½ percent and 8 percent respectively using five and ten years data) and the regression *F* statistic does a poor job in confirming the presence of seasonality.

The third set of results (those for diffuse seasonality) are in some respects the most disturbing. The results in the no seasonality case suggest discarding the dummy variable estimates when the estimates fail to reject the hypothesis of no seasonality. Yet that rule would often lead to an estimate of zero seasonality in cases of diffuse seasonality. Finally, note that the *R*^2^ statistics in short samples tend to attribute much more explanatory power to seasonality than it actually has, as becomes apparent when the sample size increases. The seemingly high degree of explanation obtained in short samples is entirely spurious in the “no seasonality” experiments and largely so in the other two experiments.

The biases obtained using the MAD procedure are similar to those using the stochastic trend model (Table 2). They are slightly higher when there is no seasonality, slightly lower with clear seasonality, and virtually the same when seasonality is poorly defined. The notable difference is that the MAD estimates exaggerate the statistical significance of the results. For large samples, the *R^2^* statistics converge to the values obtained with the stochastic trend model, though they are systematically higher for shorter samples. Second, in the case of no seasonality, exactly 5 percent of experiments should reject the hypothesis of no relationship. Instead, absence of seasonality is rejected in around 8 percent of cases using the MAD procedure (compared with 5 percent using the stochastic trend model) (Table 1, Table 2, column 3). This indicates mild over-sizing, arising from autocorrelation in the error terms generated by the moving average transformation.

Overall, three conclusions emerge. First, on model choice, the stochastic trend model is slightly preferred. It is more reliable in its statistical inference. It is also more parsimonious in its data use, which the analysis above has abstracted from.^15^ Second, on the matter of whether there is seasonality or not, if a standard *F* test rejects the hypothesis of no seasonality, one can be confident that the data are seasonal even if the gap measure will tend to be too high. If, when using a short sample, the test fails to reject the hypothesis of no seasonality it will be difficult to know whether this is because the data are not seasonal or because the test lacks power to reject that. (Tests for the significance of the seasonal factors are correctly sized when the stochastic trend model is estimated, though they lack power when the sample size is short.) Third, on the extent of seasonality, the empirical monthly dummy based estimated range measure of the seasonal gap tends to exaggerate the extent of seasonality on samples of the typical size available in Africa (5–15 years). The upward bias is larger the shorter the sample and the less well defined the seasonal pattern. Given that long monthly price time series will not be generally available in the foreseeable future (including for many other seasonal phenomena), there are important gains from procedures that can mitigate the estimated bias.

## 4 More parsimonious models

The dummy variable approach to measuring the seasonal gap is highly parametrized. This has the advantage that it does not pose many restrictions on the data, but it comes at the expense of having to estimate a large number of parameters (eleven with monthly data). The alternative is a more parsimonious seasonality model which exploits the fact that seasonality in agricultural markets is generated by the crop cycle. By imposing a harvest-based pattern on the pattern of monthly seasonality factors, parsimonious seasonality models reduce the influence of any single monthly mean price. Consequently, there is a much lower probability of an incorrect peak-trough identification (for example through an error of a single month in either direction). Intuitively, with *Y* years of data (say *Y* = 10), there are in essence only 10 observations from which each monthly effect is estimated in the monthly dummy regression (despite there being 120 price data points). In contrast, by smoothing out the variation through the imposition of a tighter parametric structure, the degrees of freedom increase and so does estimation efficiency.

Nevertheless, parsimonious specifications have a cost. The gap estimates should be more accurate so long as the actual seasonal structure conforms to the imposed structure. But if the actual structure differs, the estimates will be misleading and the gap estimate may be less accurate than the biased estimate from the dummy variable model. We consider two alternative parametric specifications. The simplest is trigonometric seasonality – in which the seasonal pattern is defined by a pure sine wave. The simplest two parameter sinusoidal trigonometric seasonality representation is

(11) $$s_{m}=\alpha \cos\left(\frac{m\pi }{6}\right)+\beta \sin\left(\frac{m\pi }{6}\right)$$

With trending data, the estimating equation is

(12) $$\Delta p_{\mathrm{ym}}=\gamma +\Delta s_{m}+u_{\mathrm{ym}}=\gamma +\alpha \Delta \cos\left(\frac{m\pi }{6}\right)+\beta \Delta \sin\left(\frac{m\pi }{6}\right)+u_{\mathrm{ym}}$$

Eq. (12) is estimable by least squares. The seasonal factor *s_m_* may be re-expressed as a pure cosine function:

(13) $$s_{m}=\lambda \cos\left(\frac{m\pi }{6}-\omega \right)$$

where $\lambda =\sqrt{\alpha^{2}+\beta^{2}}$ and $\omega =\tan^{-1}\left(\frac{\alpha }{\beta }\right)$. The parameter λ measures the amplitude of the seasonal cycle and implies a seasonal gap of 2λ. If the specification is valid, least squares estimation of Eq. (11) yields unbiased and consistent estimates of the α and β coefficients in Eq. (12). However, the implied seasonal gap 2λ is a nonlinear non-negative function of these estimates and will therefore also be biased upwards. Ghysels and Osborn (2001) provide a general discussion of trigonometric representations of seasonality.^16^

**Fig. 1:**
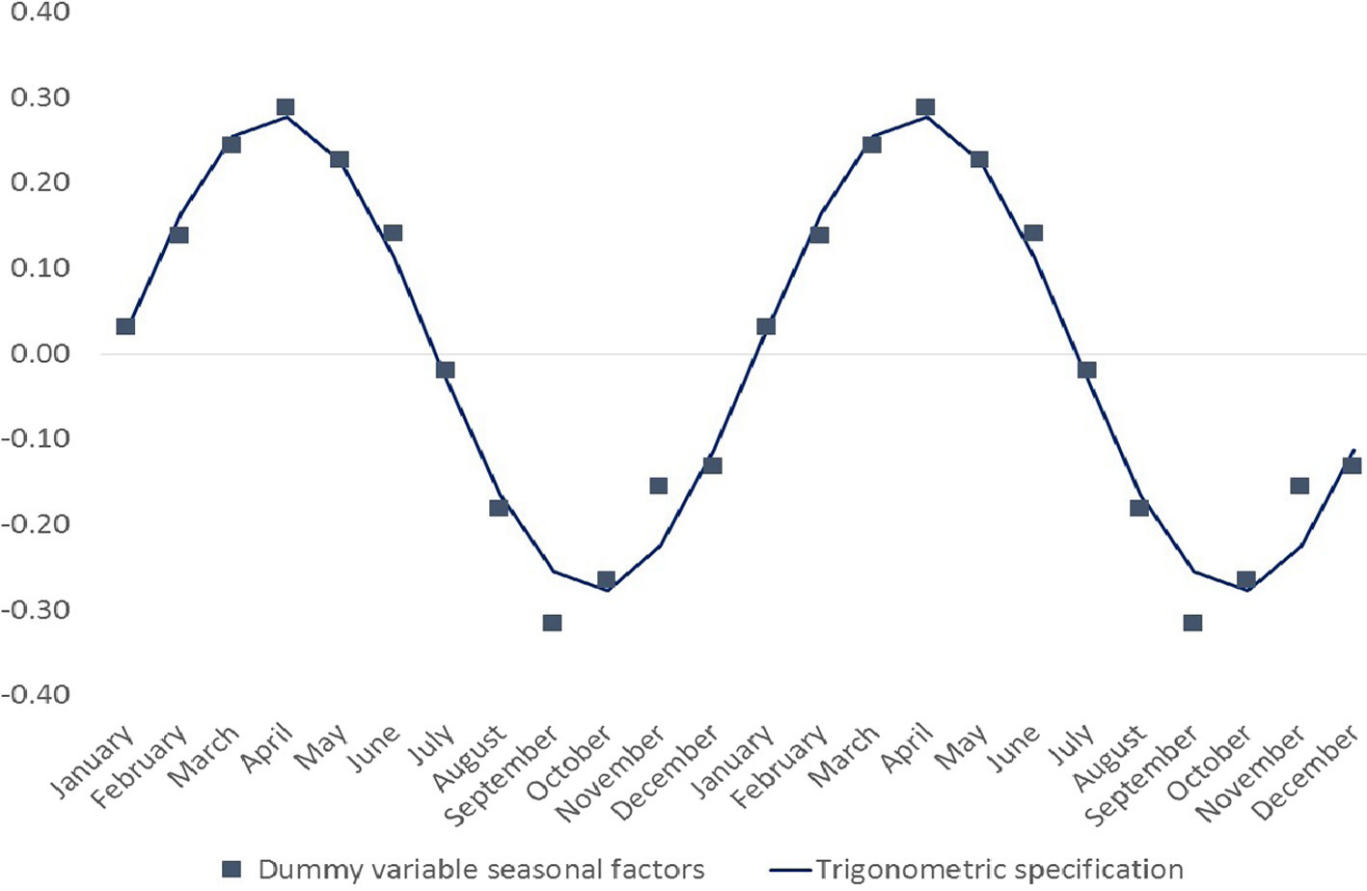
Tomato price seasonality, Morogoro, Tanzania.

The trigonometric approach is illustrated by comparing the estimated seasonal pattern with the estimated dummy variable coefficients for tomato prices in Morogoro, a provincial capital in central southern Tanzania – see Fig. 1. Tomatoes, which are annually cropped and perishable, tend to exhibit acute price seasonality and therefore provide good illustrations of seasonality profiles. This is a case in which seasonality is high and well-defined so the dummy variables procedure also works well. The estimated seasonal gap is 56% using the trigonometric specification, but 60% on the basis of the dummy variable estimates.

Although, the trigonometric specification is parsimonious, it is restrictive in that the post-harvest price decline is symmetric with respect to the pre-harvest price rise. In practice, for many crops, prices drop more rapidly post-harvest than that they rise in the remainder of the crop year. An alternative parametric specification is a sawtooth function in which prices fall sharply post-harvest and then rise at a steady rate through the remainder of the crop year – see Samuelson (1957) and, for an application, Statistics New Zealand (2010). Suppose the peak seasonal factor of λ occurs in month *m*^∗^ and that the price falls by the seasonal gap of 2λ to –λ in the harvest month *m*^∗^ + 2. The seasonal factor then rises steadily by an amount $\frac{\lambda }{5}$ over the reminder of the year. Conditional on knowing the peak price month *m*^∗^, the amplitude parameter λ may be estimated from the regression

(14) $$\Delta p_{\mathrm{ym}}=\gamma +\Delta s_{m}+u_{\mathrm{ym}}=\gamma +\lambda \Delta z_{m}(m^{*})+u_{\mathrm{ym}}$$

Here $\Delta z_{m}(m^{*})$ is equal to −1 if $m=m^{*}+1$ or $m=m^{*}+2$ and $\frac{1}{5}$ otherwise. We estimate by performing a grid search choosing the value for *m*^∗^ which gives the maximum *R*^2^ fit statistic.^17^

**Fig. 2:**
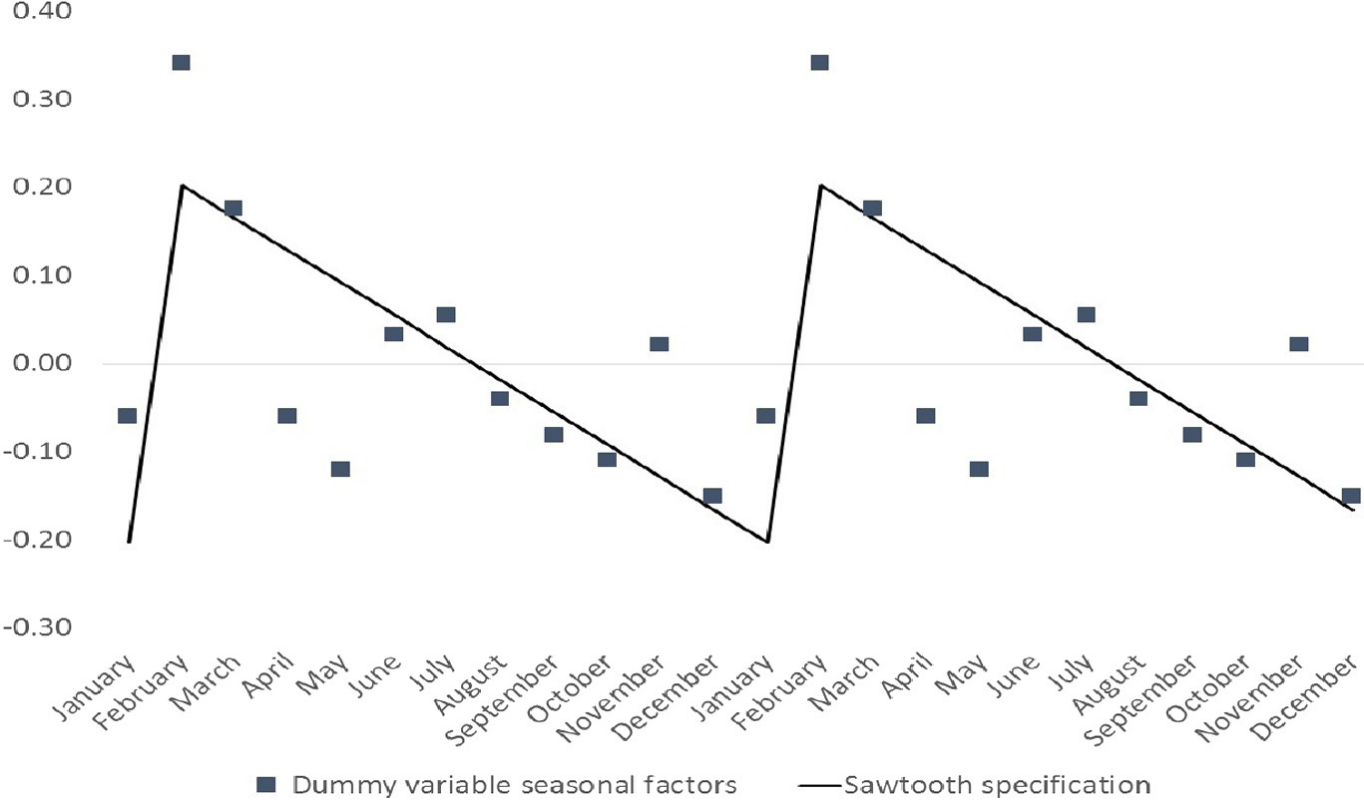
Tomato price seasonality, Lira, Uganda.

Fig. 2 illustrates a sawtooth seasonal pattern for tomato prices in Lira, an administrative center in northern Uganda. The estimated seasonal gap is 40 percent, again somewhat lower than the 52 percent using the dummy variables model.

**Fig. 3:**
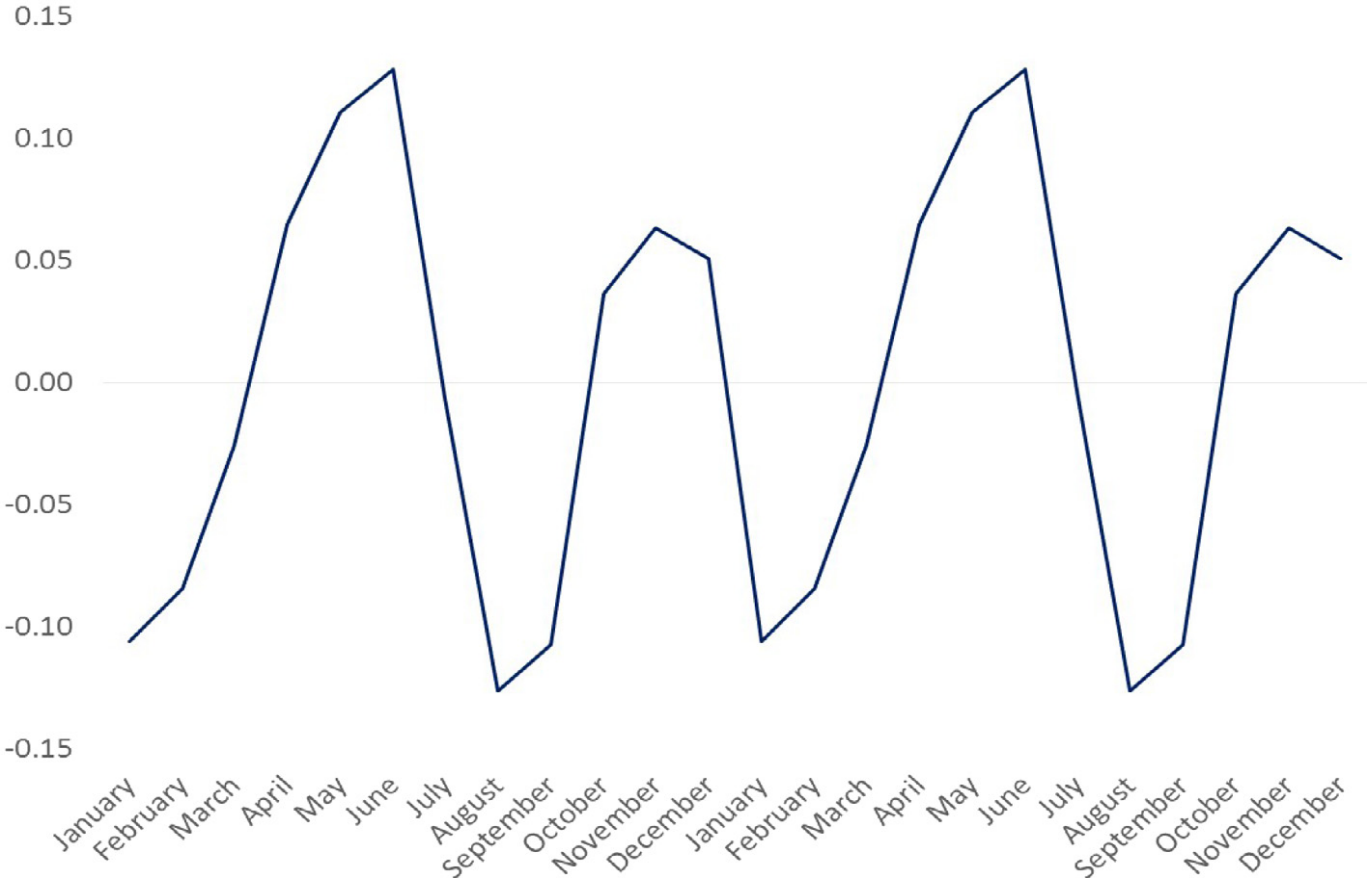
Maize price seasonality, Kampala, Uganda.

Different seasonal specifications perform better in different circumstances. The trigonometric and sawtooth specifications both suppose a single annual harvest. Fig. 3 illustrates the dummy variable seasonality estimates for wholesale maize in the Uganda capital, Kampala. Close to the equator, Kampala benefits from maize from two annual harvests – in January (17% peak to trough gap) and July (25% peak to trough gap). Neither the trigonometric nor the sawtooth models are able to account for this pattern.

We repeated the Monte Carlo experiments reported for the dummy variables and MAD estimators in Section 3. The results for the trigonometric estimator are reported in Table 3 and those for the sawtooth estimator in Table 4. When there is no seasonality in the process under investigation (left hand block), the bias falls by about 40% for trigonometric estimator and 25% for the sawtooth estimator. When there is clear seasonality (second blocks), the sawtooth estimator eliminates almost all the bias while the trigonometric estimator shows only a small (and negative) bias. Given that the data in this example were generated by a sawtooth process, it is unsurprising that the sawtooth estimator has the superior performance. The negative bias in the trigonometric process arises from the fact that the sinusoidal functional form imposes smooth peaks and troughs whereas the data generating process is spiked. The ranking would be reversed if we had used a trigonometric seasonal process to generate the data.

The third block of statistics relates to a poorly defined seasonal process. This may be the most realistic in practical applications. Both estimators generate substantial bias reductions relative to the dummy variables procedure – reductions of the order of 70 percent for the trigonometric estimator and 50 percent for the sawtooth estimator. With short data samples on poorly defined seasonal processes, the greater parsimony of these estimators leads to more reliable estimation. However, even with samples as long as 40 years, statistical significance tests have low power against the hypothesis of no seasonality – see the final column in each of Table 3, Table 4.

In summary, parsimonious seasonal models are likely to be preferable to the standard dummy variable procedure for estimating the extent of seasonality when data samples are short or seasonal processes are poorly defined. These are typical circumstances in data on prices for developing country food crops. These procedures substantially reduce the bias resulting from use of dummy variable estimators of the seasonal gap. Significance tests on the presence of seasonality remain correctly sized (i.e. they incorrectly reject the hypothesis of no seasonality in the expected proportion of cases) but they may have low power (they fail to correctly reject the hypothesis of no seasonality in a large proportion of cases). Their limitation is that they will perform poorly for crops in which there are two harvests per year.

## 5 Seasonality in African food crop prices

The extent of seasonality in food prices is examined for seven African countries: Burkina Faso, Ethiopia, Ghana, Malawi, Niger, Tanzania and Uganda. Monthly price series for 13 crops and food products in local markets over the period 2000–2012 were obtained from national statistical offices and from a private marketing agency in Uganda. The crops covered the main staple cereals (maize, millet, rice, sorghum and teff) together with cassava and a number of important fruits and vegetables, as well as eggs. The number of markets varies across countries. In four countries (Burkina Faso, Niger, Tanzania and Uganda), prices are reported both at the retail and wholesale level, although not always for the same marketplaces. For the other three countries there are only wholesale prices. This dataset yields a total of 1053 location-food crop pairs. Table 5 provides more detailed information.

Prices are all expressed in nominal terms and local currency. There has been substantial inflation during the sample period in some of the countries. Deflation of the price of a major food staple by the local CPI would, however, remove part of the variation of interest. We rely on the trend in Eq. (1) to account for the impact of inflation and other trend-associated factors. Estimation is based on the stochastic trend model defined by Eqs. (9), (12), (14), depending on the seasonal specification.^18^

For some of the series, missing data points are a potential problem. These take two forms. Some series start later or finish earlier than others. With thirteen years of data, there will be a maximum of 156 data points in each series. We only have this full number of observations for wholesale prices in Uganda and (with some exceptions) Tanzania - see Table 5. Sample start and end dates therefore differ across series. The more serious problem is gaps within the series. This is most acute in the Burkinabe retail price series, where nearly one in five intermediate data points are absent.^19^ In those cases in which gaps are present, we use the skip estimation procedure defined by Eq. (10).

The stochastic trend model is applied to estimate the seasonal gap and a three step procedure is followed to identify the appropriate specification (dummy variable, trigonometric, or sawtooth). In the absence of precise information for all crop-location pairs on the existence of multiple growing seasons (and the exact month of harvest), it is a priori not clear whether parsimonious models are preferred over the dummy variable model, nor which of the two parsimonious models is more appropriate. Overall, the trigonometric and sawtooth gap estimates have correlations of 0.94 and 0.92 with the dummy variable estimates and 0.8 with each other. More particularly,

(a) The estimates of the trigonometric and sawtooth specifications, which are nested within the dummy specification (see Section 4), are compared against those of the dummy variable model. If the *F* test rejects both models, the dummy variables estimates are retained.
(b) If the *F* test rejects one but not both of the parsimonious procedures, the non-rejected parsimonious model is taken as an acceptable simplification of the dummy procedure, reducing bias in the seasonal gap estimates.
(c) Finally, if the *F* test fails to reject the trigonometric and sawtooth model, one of them is selected based on fit, as measured by the *R*^2^ statistic.

Using this rule, the dummy variables specification is preferred in 168 instances, the trigonometric specification in 625, and the sawtooth specification in the remaining 260. The trigonometric and sawtooth specifications are quite similar and there is little pattern in whether one or the other gives the better fit. We only adopt the dummy specification if both are rejected against the dummy alternative. This will happen if two conditions are satisfied – the seasonal pattern must be well defined and is not well reflected in a sinusoidal or sawtooth pattern. A two harvest pattern meets these two requirements but there can be other instances. Many of the cases in which the dummy specification is preferred relate to Uganda (beans, maize, matoke, oranges and tomatoes), an equatorial country where double cropping is possible for many crops. There are relatively few instances in which this specification is preferred for cassava, millet and sorghum where seasonal patterns are less well defined – see Table 6, column 3.

Based on these preferred specifications for each commodity-location pair crude seasonal gaps are calculated in the wholesale and retail markets and averaged by crop across all locations in the country (Appendix Table A1, Table A2). We report the proportion of cases in which the seasonality is statistically significant (i.e. null hypothesis of no seasonality rejected at the 5% level) in parentheses. These tests are correctly sized and a high proportion of locations in which seasonality is significant can be taken as an indication of the existence of seasonality.^20^ Yet, potential overestimation of the extent of that seasonality cannot be fully excluded, especially for commodity-locations pairs where samples are short. The predominant use of parsimonious specifications helps mitigate against such bias.

Because the sample size mainly varies by country (Table 5), the seasonality estimates for the different commodities can be partially purged from potential overestimation by regressing the 1053 estimated gaps for each commodity-location pair on the commodity type, the nature of the market (retail/wholesale), and a set of country dummies.^21^ The average estimated seasonal gaps for each commodity are reported in Table 6 (controlling for the nature of the market and country effects), together with the share of locations in which the null of no seasonality is rejected.

Fruits and vegetables (tomatoes, plantain and oranges) display the highest seasonal gaps (60.8, 49.1 and 39.8 percent respectively). This is intuitive, especially for tomatoes and oranges. They are highly perishable and their production is season-bound. Cassava and eggs, which are produced throughout the year, are among the commodities with the lowest seasonality. Furthermore, cassava can be stored underground and harvested throughout the year, as needed. The high seasonal gap for plantain (and also bananas), which are also perennials, is somewhat surprising from this perspective. Yet substantial price seasonality could still ensue, for example if their consumption is mainly countercyclical (high when other staple foods are expensive and low when they are cheap) and storage is difficult.

Among the cereals, maize shows the highest seasonal gap (33.1 percent on average), and rice the lowest (16.6 percent). Seasonality is significant in the vast majority of the markets in both cases, confirming the existence of seasonality. Moreover, with peak prices across locations on average 33.1 percent higher than during the trough, seasonality in maize prices is substantial, and about twice as high as this of rice, whose seasonal gap is estimated at 16.6 percent. Higher seasonality of maize among the cereals could also be expected, given lower storability and greater post-harvest loss than millet and sorghum (World Bank, 2011). With Africa a growing importer of rice (which is becoming more important in the urban diets), rice markets are more closely linked with the international markets. Part of African rice production is also irrigated. The other cereals (teff, sorghum, millet) have seasonal gaps of around 20–24 percent. They tend to store better—they have smaller grains and are cultivated in dryer areas. On average, seasonal gaps are 3.4 percent higher in wholesale than in retail markets. This is in line with experience in developed economies where a substantial proportion of the value of retail products is generated by transport costs and by labor costs in retailing.

Table 7 further shows the estimated country effects, with the caveat that they reflect both the country effects and potential short sample bias.^22^ Niger, Burkina Faso, Malawi and Ghana are associated with the highest average seasonal gaps, all in excess of 30 percent at the wholesale level. Ethiopia has the lowest average gap at approximately 15 percent. Tanzania and Uganda, which also have the longest samples, are intermediate at around 25 percent. The findings for Niger and Burkina are intuitive and consistent with other studies.^23^ Dryland agriculture is predominant in both countries and the raining season short (and erratic). The large gap observed in Ghana is less expected, however, and may be related to the short duration of the price series (only 6 years at most) implying higher potential bias. Ghana also displays the largest proportion of locations where its seasonality is not statistically significant (Appendix Table A1, Table A2).

Seasonal gaps measure the extent of seasonality. A second question posed in the introduction was that of the share of monthly price variation attributable to seasonality. This share is measured by the seasonal *R*^2^ which is simply the standard regression *R*^2^ in Eqs. (9), (12), (14), depending on the specification. Among crops, plantain/matoke and maize show the largest (0.32 and 0.25 respectively) and cassava and cowpeas the lowest seasonal *R*^2^s (0.08 and 0.09 respectively) – see Table 6. Across countries seasonality appears to explain around 17 percent of overall price variability.^24^ It increases to 27.7 and 21.3 percent in Niger and Burkina Faso respectively, where agriculture is also mainly rain-fed and highly seasonal. While the bulk of intra-annual price variability is not related to seasonal fluctuations, for a number of crops (maize) and countries (especially in the Sahel), its contribution appears nonetheless non-negligible.

Thirdly, we ask whether the seasonality we find in African food markets is excessive? Some seasonality in prices is to be expected when production is seasonal, given storage costs. But what should count as excessive? Most of the products considered are non-traded in the sense that only small quantities cross national borders. However, this is not true of either maize or rice and for these two commodities the national seasonal gaps can be compared with those on the relevant international market. White maize predominates in human consumption through most of Africa rather than the yellow maize typically consumed in the developed world. The Johannesburg futures market (SAFEX) provides the reference price for white maize in southern and east Africa. This price is quoted in rand. For rice, the most commonly used reference price is the Bangkok spot price (5 per cent broken) which is quoted in US dollars. In both cases, we use monthly prices over the 13 year period 2000–12.^25^

Seasonality is well defined in both price processes, with the dummy variables specification preferred in each case. The estimated seasonal gaps are 12.2 percent for SAFEX white maize and 5.1 percent for Bangkok rice.^26^ These statistics are to be compared with the average maize and rice seasonal gap estimate of 33.1 percent and 16.6 percent respectively. In both cases, seasonality is on average two and a half to three times as acute in local African markets as on the relevant international market. Moreover, the local African seasonal *R*^2^ coefficients for maize and rice are an order higher than those for the corresponding world markets (6.0 percent for SAFEX maize and 2.2 percent for Bangkok rice).

**Fig. 4:**
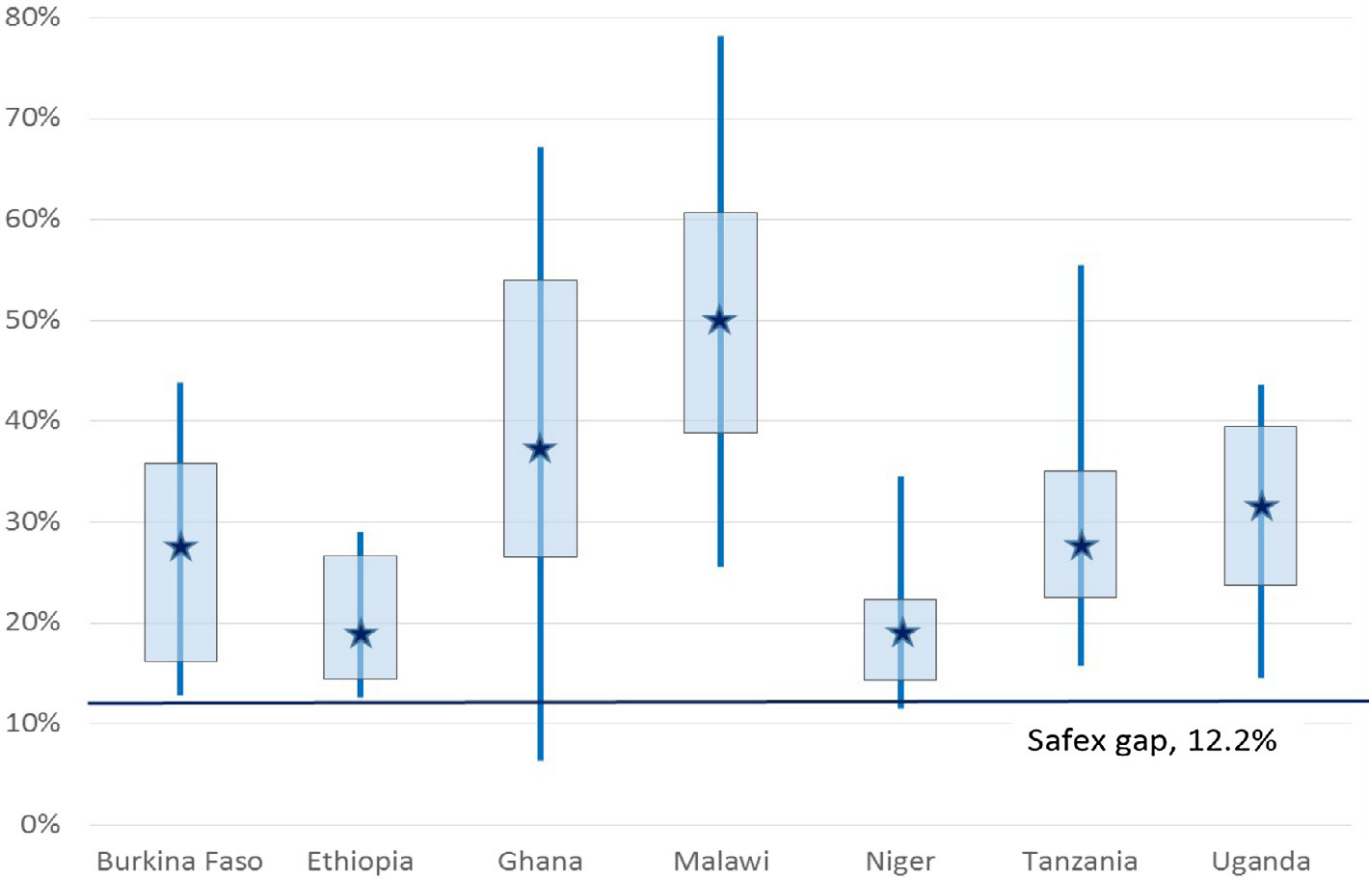
Seasonal gaps for wholesale maize.

**Fig. 5:**
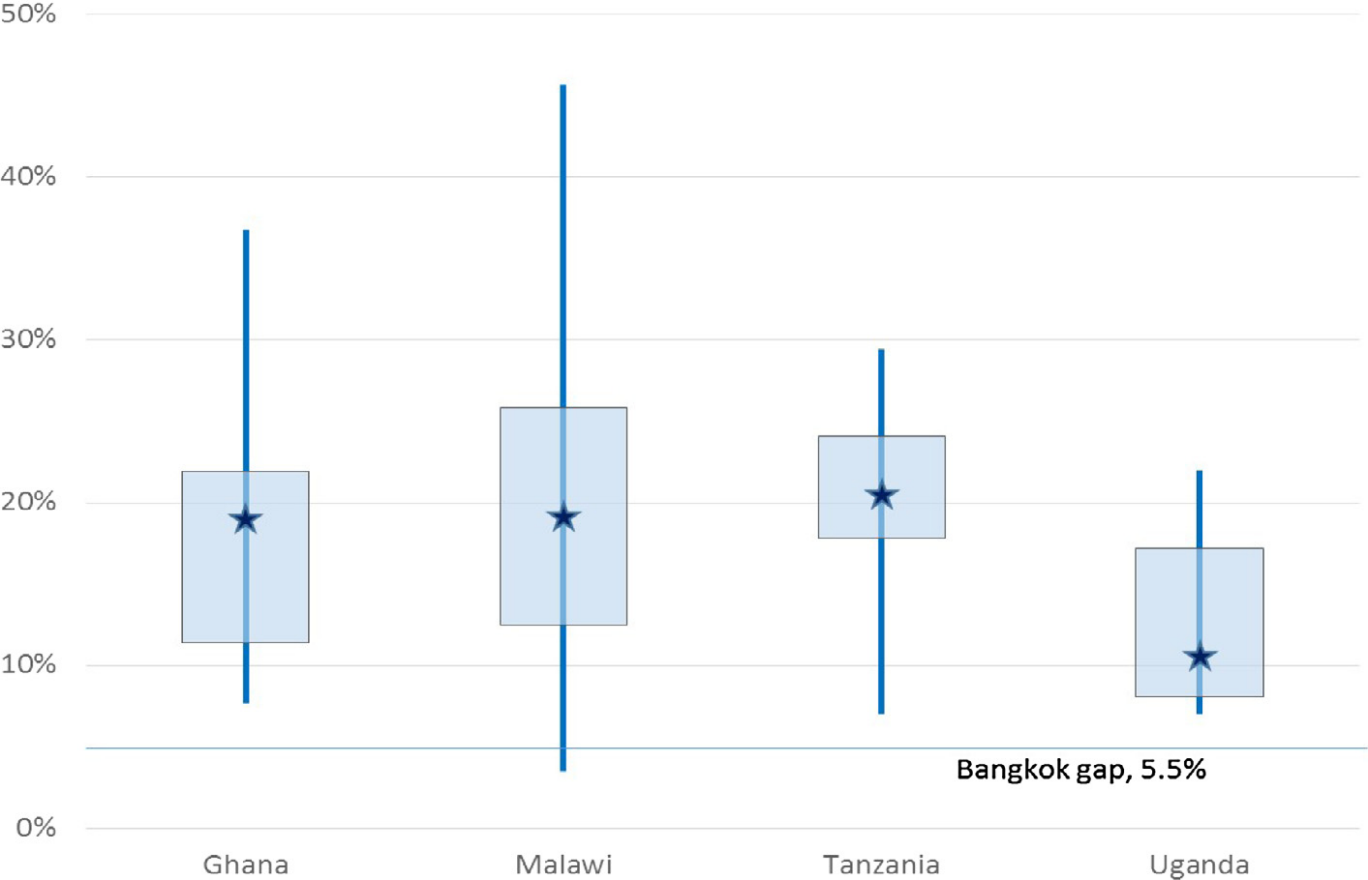
Seasonal gaps for wholesale rice.

Fourth, how widespread is excess seasonality? Fig. 4, Fig. 5 give a visual summary of the maize and rice seasonal gap distributions in each of the seven countries relative to the respective international reference market. The vertical lines measure the range of seasonal gaps across markets in each country, i.e. the distance between the largest and smallest gap, while the rectangles demarcate the interdecile range between the 20 percent and 80 percent points in the gap distribution. Seasonality is larger than in the international reference market in virtually all of the 133 wholesale maize and 107 wholesale rice markets examined. There are only two centers where the estimated gap for maize is lower than the SAFEX gap of 12.2 percent (Ho in Ghana and Niamey in Niger) and three where the gap is lower than the 5.1 percent gap in the Bangkok spot market for rice (Santhe, Lizulu and Neno, in Malawi). The occurrence of excess seasonality is widespread. Nonetheless, there is also substantial variation in the extent of seasonality across locations within countries, as in Malawi, Ghana and Tanzania (for both maize and rice). This counsels caution against overgeneralization from case studies and underscores the need for differentiated and targeted interventions.

Fifth, at 50.6 percent on average (Table A1), maize price seasonality is particularly striking in Malawi. Households appear to suffer a double seasonality impact – the main staple food is maize which has the highest seasonal gap among the cereals (Table 6) and there is a large country effect (Table 7). From this perspective, the attention in the seasonality literature to this specific country-crop pair does not surprise– see for example Manda, 2010, Chirwa et al., 2011 and Ellis and Manda (2012).

But is the Malawi effect an exception? Put differently, to what extent is the variability of seasonal gaps affected by national boundaries (to be distinguished from overall country effects). An analysis of variance exercise, reported in Table 8, casts light on this question. This shows that 30.4 percent of the variation of the preferred seasonal gap measure is attributable to the crop, 14.5 percent to the (market) location and only 0.5 percent to the country and 0.4 percent to the market level (wholesale or retail). Country-specific variation is not statistically significant.

Looking at each crop separately we find statistically significant country differences only for maize and plantain, while most seasonality is attributable to market location.^27^ This suggests that differences in seasonality arising from geographical location are likely to be caused more by transport factors than by differences in government policies at the national level, the single important exception being for maize where, controlling for the market level (wholesale versus retail) the Malawian seasonal gap averages 23 percent higher than the average of the other countries.

We performed two exercises in order to further explore the Malawian maize seasonal gap. First, using real local maize prices for Malawi spanning 23 years (1989–2012) instead of 8 (2005–2012), the preferred seasonal gap estimate is 39.5 percent (instead of 50.6 percent, Table A1).^28^ The lower figure may in part reflect the use of deflated prices. Irrespectively, it remains higher than the wholesale estimates for all the other countries in our sample. Second, we compared the maize gaps across locations on both sides of the Malawian and Tanzanian borders where cultivation and meteorological conditions are similar. Mbeya, which is the capital of the Tanzanian region (*mkoa*) of the same name contiguous with the border with Malawi, exhibits a maize seasonal gap of 22.8 percent. Chitipa (143 km from Mbeya, maize seasonal gap 48.3 percent), Karonga (161 km, 74.8 percent) and Misuku (180 km, 71.6 percent) are the closest locations on the Malawian side of the border.^29^ Maize price seasonality appears to change dramatically over these relatively short cross-border differences.

The prevalence of high seasonal gaps throughout Malawi together with the sharp drop in the gap as one moves north into Tanzania suggests that the high Malawian gaps are the results of political or institutional factors specific to the country rather than agroeconomic factors. To that extent, it should be possible to reduce some of the more extreme instances of seasonal price variation in Malawi, including by facilitating cross-country trading, which would also benefit Tanzania.^30^

## 6 Concluding remarks

As development practitioners and economists, we are all well aware of seasonality in African livelihoods, much of it originating from seasonality in food prices. At the same time, it is unclear what exactly it is we know. The issue has somewhat disappeared to the background during the 2000s and an updated and systematic review of its extent, especially in the African context, has been missing. In addition, most of our empirical knowledge has been based on very short samples and (purposively sampled) case studies, often confounding intra-annual variation with seasonality and generalizing from a non-representative base.

This paper has contributed to extending what we know about seasonality, both by revealing some of the shortcomings in the standard practice of measuring it, as well as by systematically examining the extent of price seasonality in Africa using a uniform methodological approach. In total, the seasonal price gap was estimated across thirteen staple and non-staple crops/products in seven countries from across southern, eastern and western Africa during the 2000s and early 2010s, yielding a total of 1053 location-commodity pairs. Five key insights emerge, with important implications for further empirical work and policy orientation.

First, on methodology, the simple, most widely used (monthly) dummy variables and moving average deviation approaches to measuring the seasonal price gap overestimate the extent of price seasonality. This holds especially when the samples are short (up to 15 years), when the peak and trough months are not known a priori and when the seasonal pattern is either unclear or absent. With short samples of data, the trigonometric and sawtooth models, which are less flexible, but more parsimonious, can produce substantially more accurate estimates of the seasonal gap (8–9 percent lower than those found using the dummy variable model, as illustrated through Monte Carlo simulations). Caution is warranted in using dummy variable models in future empirical work to estimate the seasonal gap, especially when less than 15 years of (monthly) price data are available and the peak and trough months are not a priori known.

Second, turning to the findings, food price seasonality in Africa remains substantial (despite the somewhat lower estimates than those reported in the literature). It is also quite diverse across crops, regions and market places. Looking across commodities and countries, the average seasonal gap is 28.3 percent. It is highest for fruits and vegetables (60.8 percent for tomatoes) which are highly perishable and whose production is seasonal, and lowest for eggs and cassava, which are harvested throughout the year and which can be stored in the ground (cassava). Among the cereals, seasonality is highest for maize (33.1 percent), about half this for rice (16.6 percent), which is more irrigated and traded internationally, and around 20–24 percent on average for millet, sorghum and teff (which store better than maize). These averages hide substantial differences across markets within countries, cautioning against generalizations from non-representative samples, and highlighting the need for targeted interventions (e.g. when providing better storage facilities).

Third, African seasonal price variability appears substantially higher than this observed internationally. Looking at both maize and rice, for which there are well-defined international reference prices, the seasonal price gap is two and a half to three times higher than on the international reference markets. This suggests substantial scope for reduction.

Fourth, price seasonality explains on average about 17 percent of domestic staple crop volatility, rising to 25 percent for maize. Clearly, domestically, there is substantial regularity in price volatility, especially for maize. Internationally, the share is only 6 percent.

Finally, seasonal gaps vary more according to the identity of the crop than the location at which the price is measured. Looking at each food crop separately, there is only evidence of statistically significant country-specific variation in seasonal gaps for maize and, to a lesser extent, plantain. Specifically, Malawi stands out as having the highest maize seasonal gaps both in terms of statistical criteria and in cross-border comparisons with neighboring locations in Tanzania.

Together these findings indicate that the current neglect of seasonality in the policy debate is premature. First, the results underscore the importance of correcting for seasonality in food prices when constructing welfare and poverty measures, a largely ignored issue among poverty measurement practitioners so far – see Muller (2002) and Van Campenhout et al. (2015). Second, they suggest important welfare losses for the large share of (often poor) net food buying households even in the rural areas, where they frequently engage in sell-low, buy-back high behavior (Stephens and Barrett, 2011, Palacios-Lopez et al., 2015). Third, this suggests important gains from better post-harvest storage techniques through exploitation of the seasonal price differentials (Gitonga et al., 2013).

Whether food price seasonality also translates into seasonal declines in the quantity and quality of diets and nutritional outcomes, will depend on a series of other factors such as the substitutability among crops, the net marketing position of households, their access to financial markets, and their capacity to store crops. Establishing the link between price and consumption seasonality was beyond the scope of this paper. Yet the levels of staple price seasonality documented here, the recent reconfirmation of continued seasonality in African diets (Savy et al., 2006, Becquey et al., 2012, Hirvonen et al., 2015) and the adoption of the Zero Hunger goal provide an important impetus as well as building blocks for further research into these topics.

## Figures and Tables

**Table A1:** Seasonal gap estimates and statistical significance (wholesale markets).

	Burkina Faso	Ethiopia	Ghana	Malawi	Niger	Tanzania	Uganda
Bananas (sweet)		9.1% (20%)	48.6% (54%)				
Beans				27.7% (77%)		23.2% (90%)	28.1% (100%)
Cassava			19.2% (8%)	26.6% (28%)			20.1% (50%)
Cowpeas			14.5% (7%)		47.5% (100%)		
Eggs		14.3% (100%)	7.2% (36%)				
Maize	26.9% (56%)	19.8% (100%)	38.0% (71%)	50.6% (100%)	20.1% (100%)	29.4% (100%)	31.1% (88%)
Matoke/Plantain			61.5% (69%)				28.8% (63%)
Millet	23.4% (64%)		9.2% (21%)			20.2% (23%)	19.0% (75%)
Oranges		21.0% (51%)	33.6% (46%)				
Rice			18.4% (15%)	19.9% (73%)		19.9% (85%)	12.5% (75%)
Sorghum	24.7% (45%)	13.6% (80.0%)	11.8% (21%)			14.5% (23%)	22.6% (100%)
Teff		10.3% (100%)					
Tomatoes		36.3% (73%)	98.0% (57%)				

The table reports averages of the seasonal gap estimates for each country-commodity pair in wholesale markets irrespective of statistical significance using the preferred gap estimates Numbers in parentheses show the proportion of locations for which the preferred gap estimate is based on coefficients which are significant at the 95% level.

Ethiopia: teff refers to white teff.

Ghana: rice refers to locally produced rice; plantain is ap’tu plantain; bananas are ap’em plantain.

**Table A2:** Seasonal gap estimates and statistical significance (retail markets).

	Burkina Faso	Niger	Tanzania	Uganda
Bananas (sweet)			16.1% (20%)	12.9% (25%)
Beans			12.4% (60%)	28.7% (100%)
Cassava			18.9% (20%)	10.0% (13%)
Cowpeas		41.3% (100%)		5.7% (100%)
Eggs				4.6% (13%)
Maize	62.4% (80%)		22.8% (50%)	13.5% (75%)
Matoke/ Plantain				42.1% (100%)
Millet	30.9% (95%)	28.5% (100%)	10.1% (0%)	8.6% (38%)
Oranges			37.2% (80%)	40.2% (100%)
Rice			17.3% (95%)	13.4% (75%)
Sorghum	33.2% (88%)	20.6% (100%)		40.4% (100%)
Tomatoes			42.2% (75%)	36.8% (63%)

The table reports averages of the seasonal gap estimates for each country-commodity pair irrespective of statistical significance in retail markets using the preferred gap estimates. Numbers in parentheses show the proportion of locations for which the preferred gap estimate is based on coefficients which are significant at the 95% level.

**Table 1:** Dummy variable bias.

Years	No seasonality			Clear seasonality			Poorly defined seasonality		
	Bias	*R*^2^	Statistical significance (%)	Bias	*R*^2^	Statistical significance (%)	Bias	*R*^2^	Statistical significance (%)
5	0.2126	0.1866	5.1	0.0828	0.2522	22.9	0.1425	0.1959	6.7
10	0.1504	0.0926	5.0	0.0410	0.1662	51.3	0.0843	0.1028	9.0
20	0.1062	0.0460	5.1	0.0182	0.1238	88.9	0.0459	0.0569	14.6
40	0.0752	0.0230	5.0	0.0069	0.1027	99.8	0.0213	0.0341	28.0

Estimated bias in gap estimation from dummy variables regression based on 100,000 replications. Price changes are normally and independently distributed with mean and variance equal to 0.01. The data for the estimates reported in the first block (columns 1–3) do not show any seasonality, those in the second block (columns 4–6) exhibit a clearly defined seasonal peak and trough with a gap of 20% and those in the final block (columns 7–9) show a diffuse and poorly defined seasonal pattern with a gap of 8%. *R*^2^*indicates* share of the price variation in the sample on average “explained” by the seasonal factors and the proportion of simulations in which the regression *F* statistic rejects the hypothesis of no seasonality is reported under “statistical significance”.

**Table 2:** Bias for Moving Average Deviations Procedure.

Years	No seasonality			Clear seasonality			Poorly defined seasonality		
	Bias	*R*^2^	Statistical significance (%)	Bias	*R*^2^	Statistical significance (%)	Bias	*R*^2^	Statistical significance (%)
5	0.2183	0.2001	7.8	0.0689	0.2692	29.7	0.1441	0.2104	10.3
10	0.1544	0.1003	8.2	0.0212	0.1791	59.9	0.0830	0.1116	13.5
20	0.1093	0.0502	8.2	−0.0093	0.1339	92.4	0.0415	0.0620	20.6
40	0.0772	0.0250	8.0	−0.0301	0.1113	99.9	0.0145	0.0372	36.1

Estimated bias in gap estimation from dummy variables regression of deviations from a centered moving average trend based on 100,000 replications. Price changes are normally and independently distributed with mean and variance equal to 0.01. The data for the estimates reported in the first block (columns 1–3) do not show any seasonality, those in the second block (columns 4–6) exhibit a clearly defined seasonal peak and trough with a gap of 20% and those in the final block (columns 7–9) show a diffuse and poorly defined seasonal pattern with a gap of 8%. *R*^2^*indicates* share of the price variation in the sample on average “explained” by the seasonal factors and the proportion of simulations in which the regression *F* statistic rejects the hypothesis of no seasonality is reported under “statistical significance”.

**Table 3:** Bias for the trigonometric seasonality estimator.

Years	No seasonality			Clear seasonality			Poorly defined seasonality		
	Bias	*R*^2^	Statistical significance (%)	Bias	*R*^2^	Statistical significance (%)	Bias	*R*^2^	Statistical significance (%)
5	0.1325	0.0339	5.0	−0.0082	0.0614	19.4	0.0614	0.0386	7.1
10	0.0938	0.0168	5.0	−0.0300	0.0458	37.9	0.0261	0.0216	9.5
20	0.0662	0.0083	5.0	−0.0401	0.0381	68.6	0.0037	0.0133	15.1
40	0.0469	0.0042	5.1	−0.0457	0.0343	94.8	−0.0096	0.0092	26.4

Estimated bias in gap estimation from trigonometric regression based on 100,000 replications. Price changes are normally and independently distributed with mean and variance equal to 0.01. The data for the estimates reported in the first block (columns 1–3) do not show any seasonality, those in the second block (columns 4–6) exhibit a clearly defined seasonal peak and trough with a gap of 20% and those in the final block (columns 7–9) show a diffuse and poorly defined seasonal pattern with a gap of 8%.

**Table 4:** Bias for the sawtooth seasonality estimator.

Years	No seasonality			Clear seasonality			Poorly defined seasonality		
	Bias	*R*^2^	Statistical significance (%)	Bias	*R*^2^	Statistical significance (%)	Bias	*R*^2^	Statistical significance (%)
5	0.1618	0.0641	4.9	0.0026	0.1131	31.7	0.0887	0.0699	7.1
10	0.1145	0.0318	5.0	0.0054	0.0920	67.7	0.0445	0.0380	10.2
20	0.0809	0.0158	5.2	0.0006	0.0856	96.4	0.0160	0.0228	18.5
40	0.0572	0.0019	5.1	0.0001	0.0835	100	−0.0006	0.0156	37.4

Estimated bias in gap estimation from sawtooth regression based on 100,000 replications. Price changes are normally and independently distributed with mean and variance equal to 0.01. The data for the estimates reported in the first block (columns 1–3) do not show any seasonality, those in the second block (columns 4–6) exhibit a clearly defined seasonal peak and trough with a gap of 20% and those in the final block (columns 7–9) show a diffuse and poorly defined seasonal pattern with a gap of 8%.

**Table 5:** Data availability.

		Commodities	Locations	Pairs	Start date	End date	Observations	Gaps
Burkina Faso	Wholesale	3	11	31	Jan-00	Sep-11	24–144	5.1%
	Retail	3	49	126	Jul-04	Sep-11	38–96	19.3%

Ethiopia	Wholesale	11	11	71	Jan-03	Dec-12	49–120	None
Ghana	Wholesale	11	14	149	Jul-06	Aug-11	46–68	1.9%
Malawi	Wholesale	4	68	253	Apr-05	Dec-12	26–93	11.7%

Niger	Wholesale	2	8	10	Jan-02	Dec-12	94–131	1.8%
	Retail	3	14	22	Jan-02	Dec-12	95–132	1.3%

Tanzania	Wholesale	5	20	86	Jan-00	Dec-12	27–155	5.8%
	Retail	8	20	160	Jan-02	Dec-12	33–132	0.1%
Uganda	Wholesale	7	8	56	Jan-00	Dec-12	64–156	0.8%
	Retail	12	8	89	Jul-05	Dec-12	90	None

Total	*Wholesale*	*43*	*140*	*656*				
	*Retail*	*26*	*91*	*397*				

In many countries, price data are either not reported for all commodity-location pairs or are insufficient for analysis.

The start dates and end dates reported in the table give the maximum extent of the series. The actual number of data points is less than this maximum number because of a later start, earlier finish or gaps in the series. The final column reports the overall proportion of gaps in the data series.

**Table 6:** Average estimated seasonal gap and seasonal *R*^2^ by food crop.

	Seasonal gap (%)	Seasonality significant (%)	Seasonal *R*^2^
Tomatoes	60.8	64.0	0.21
Plantain/matoke	49.1	66.7	0.32
Oranges	39.8	50.0	0.16
Maize	33.1	93.2	0.25
Bananas	28.4	39.1	0.13
Teff	24.0	100.0	0.15
Beans	22.9	81.7	0.21
Sorghum	22.0	48.2	0.15
Millet	20.1	41.3	0.16
Cassava	18.8	26.9	0.08
Rice	16.6	68.2	0.17
Cowpeas	17.6	27.8	0.09
Eggs	14.1	64.0	0.18

Average	28.3	59.3	0.17

The table reports the regression estimates of the average seasonal gap in wholesale markets, the proportion of locations for which the preferred gap estimate is based on coefficients which are significant at the 95% level and seasonal *R*^2^ by crop. The averages reported in the bottom row of the table are the unweighted averages across crops.

**Table 7:** Average estimated seasonal gap and seasonal *R*^2^ by country.

	Seasonal gap (%)	Seasonality significant (%)	Seasonal *R*^2^
Burkina Faso	33.2	54.8	0.21
Ethiopia	14.5	77.5	0.15
Ghana	31.4	36.9	0.13
Malawi	34.0	70.8	0.19
Niger	36.6	64.5	0.28
Tanzania	24.4	59.8	0.09
Uganda	23.7	65.5	0.16

Average	28.3	61.4	0.17

The table reports the regression estimates of the average seasonal gap in wholesale markets, the proportion of locations for which the preferred gap estimate is based on coefficients which are significant at the 95% level and seasonal *R*^2^ by country. The averages reported in the bottom row of the table are the unweighted averages across crops.

**Table 8:** Analysis of variance.

	Crop	Retail/wholesale	Country	Market location	Observations	*R*^2^
All	30.4%^∗∗∗^	0.4%^∗∗^	0.5%	14.5%^∗∗∗^	1053	52.2%^∗∗∗^
Beans	–	5.2%^∗∗∗^	0.6%	76.3%^∗∗∗^	121	76.3%^∗∗∗^
Cassava	–	0.3%	0.1%	91.7%^∗^	106	91.7%^∗^
Maize	–	0.7%^∗∗^	3.7%^∗∗∗^	50.8%^∗∗∗^	202	96.4%^∗∗∗^
Millet	–	0.3%	2.0%	40.2%	131	40.2%
Plantain	–	–	13.0%^∗∗^	71.0%	28	98.1%^∗^
Rice	–	0.3%	0.2%	88.5%^∗∗∗^	135	88.5%^∗∗∗^
Sorghum	–	0.5%	0.5%	51.0%^∗^	106	51.0%^∗∗^

The first row of the table reports a four way analysis of variance of the preferred measure of the seasonal gap for the complete set of food commodities analyzed in the paper (the listed commodities plus bananas, eggs, oranges, teff and tomatoes). The remaining rows report the three way analysis of variance (two way for plantain) for those commodities there is sufficient variation to calculate significance tests. In each case, the reported statistic is the proportion of the variance attributable to the factor.

^∗∗∗^, ^∗∗^ and ^∗^ indicate significance at the 99%, 95% and 90% levels respectively.

## Appendix A

See Table A1, Table A2.

## Footnotes

3 A number of country-specific studies exist but these stop short of quantifying the extent of seasonal price variation. Examples include Allen (1954), who analyzed seasonality in UK food prices over the immediate post-war decade, and Statistics New Zealand (2010), which reviewed seasonality in New Zealand fruit and vegetable prices. Recent country case studies from Sub-Saharan Africa, where food price seasonality is expected to be highest, include Manda (2010) (maize, Malawi), Aker (2012) (millet, Niger), Gitonga et al. (2013) (maize, Kenya), and Hirvonen et al. (2015) (food price index, Ethiopia).

4 http://www.un.org/en/zerohunger/challenge.shtml.

5 The need for reviving attention to the reality of seasonality in African livelihoods among development scholars and practitioners is also highlighted by Devereux et al. (2011).

6 Price seasonality may also follow from seasonal patterns in demand such as those related to festivities, e.g. high sugar demand in preparation of the Eid festival, or celebration of the New Year and Orthodox religious festivities (Meskel) in September in Ethiopia (Hirvonen et al., 2015).

7 Few studies explicitly study the link between food price seasonality and seasonality in diets and nutrition. Related studies include Chambers et al., 1981, Dostie et al., 2002, and Stephens and Barrett (2011).

8 A number of alternative measures of intra-annual price variability are available, all based on the month price means. However, these measures do not relate directly to the harvest cycle and so are better regarded as general measures of intra-annual price variability than of seasonality. This is true of both the intra-annual price standard deviation and the intra-annual Gini coefficient, both of which compare prices in every month and not just those pre- and post-harvest.

9 See Harvey (1990, chapter 1). In a large sample, one might wish to include an autoregressive component in the decomposition defined by Eq. (1) such that the disturbance term becomes innovational. In a short sample, this runs the risk of confusing the autoregressive and seasonal components. Seasonality patterns may also vary over time, either in an evolutionary manner, perhaps in relation to climate change, or randomly if harvest dates are random. These issues are important but cannot easily be examined with short data samples. The analysis presented here abstracts from time varying seasonality. For notational simplicity we suppose that the data cover complete years so that the first observation *p*_11_ represents the price in January of year 1 and the final observation *p_Y_*_,12_ is the price in December of year *Y*. For m+j>12, $p_{y\text{,}m+j}=p_{y+1\text{,}m+j-12}$ and for m-j<1, $p_{y\text{,}m-j}=p_{y-1\text{,}m-j+12}$.

10 Interpolation requires a model which will need to contain seasonal factors. This induces circularity into the gap estimation.

11 Eq. (9) can be equivalently re-expressed in terms of the undifferenced dummy variables and differenced coefficients.

12 In an earlier draft of this paper, we reported estimates in which we had interpolated over gaps in the series. These results differed sharply from those we now report in those cases in which the gaps were substantial.

13 Differencing will induce a serially correlated disturbance term. This is the same problem which arises in the MAD model where the serial correlation arises from the trend estimation procedure. Serial correlation will not result in any bias in the estimated seasonal factors but will complicate inference.

14 The maximum value of a set of numbers is a nonlinear function of the data – a particular observation, that corresponding to the maximum, gets a weight of one and all other observations have zero weight. Crucially, the identity of this maximal value is determined by the sample. The same is true of the minimum. The range, here interpreted as the seasonal gap, which is the difference between the maximum and minimum values, is a linear function of two nonlinear functions of the data and is therefore itself a nonlinear function of the data.

15 It remains that the MAD procedure uses up twelve monthly observations in estimating the trend. The estimates reported in Table 2 relay on 5, 10, 20 and 40 years of detrended data equivalent to 6, 11, 21 and 41 years of raw data. The close correspondence in the two sets of Monte Carlo results is ensured by use of a common random number seed in the two sets of experiments.

16 The dummy variable model, which contains 11 parameters, can be expressed in terms of six sinusoidal functions as in Eq. (12) with frequencies of 12, 6, 4, 3 and $\frac{12}{5}$ months respectively – see Ghysels and Osborn (2001). This representation also contains 11 parameters. Eq. (12) restricts 9 of these parameters to zero. It follows that the trigonometric Eq. (12) is nested within the dummy variables Eq. (6) and can be tested against it using a standard *F* test.

17 Strictly, if the peak month *m*^∗^ is estimated, Eq. (14) is not nested within the dummy variables representation (9). If *m*^∗^ were known, it would be nested and impose 10 restrictions on the dummy variables coefficients in Eq. (9). In what follows, we perform F tests against the dummy variables specification as if the two equations were estimated but adjust the degrees of freedom associated with the sawtooth representation to obtain correctly sized tests under the null hypothesis of no seasonality. See Meyer and Woodroofe (2000). Monte Carlo experiments led us to associate 4.1 degrees of freedom with the specification in Eq. (14). This number is reflected in the results reported in column 3 of Table 4 (below).

18 In Kaminski et al. (2016) we apply these methods to deflated Tanzanian maize and rice data using slightly longer samples. The results are comparable to those reported here.

19 We dropped a number of series from the analysis on the basis of insufficient data. We require (a) at least 24 observations, with (b) at least one observation for each month (otherwise the dummy variables estimator ceases to be identified) and (c) a maximum of 50% missing intermediate (gap) observations. We also dropped both the wholesale and retail rice price series for Niger – these only changed intermittently, suggesting that they might be administered or official prices.

20 That said, a high rejection rate of seasonality may follow also from small sample size (false negatives).

21 We also experimented by adding a variable measuring the number of observations available for the estimation of the seasonal gap. This variable is correlated with the country dummies making it difficult to extract country effects. The results reported here omit this sample size variable.

22 The average statistics reported in the final row of Table 7 may differ from those in the final row of Table 6 because of differences in the number of reporting locations in each country and for each food crop.

23 Using simple monthly averages of the 1996–2006 real prices, Aker (2012) also reports a high seasonal gap of for millet in Niger (44%).

24 These statistics may show an upward bias in small samples (Section 3), though the country fixed effects also helped control for this.

25 Sources: Johannesburg Stock Exchange, https://www.jse.co.za/downloadable-files?RequestNode=/Safex/PriceHistory/Spot%20Months%20on%20all%20Grain%20Products and IMF, *International Financial Statistics*.

26 In Kaminski et al. (2016) we used a slightly different model selection criterion which favored the dummy specification for these two price series. This gave slightly narrower seasonal gaps.

27 This analysis requires data at both the retail and the wholesale level for at least one country. This prevents our performing this analysis for bananas, eggs, oranges, teff and tomatoes. For maize, we have gap estimates for all seven countries under analysis while for plantain information is confined to Ghana and Uganda.

28 The price data are from the dataset used by Dana et al. (2006). Given rapid inflation in Malawi during the 1990s, prices were deflated by the CPI (IMF, International Financial Statistics).

29 Quoted distances are the distances for the fastest road connections.

30 For Tanzania, Baffes et al. (2015) demonstrate for example an adverse effect on its own maize markets of its intermittent imposition of its export bans.
